# *MET* Gene Copy Number Predicts Worse Overall Survival in Patients with Non-Small Cell Lung Cancer (NSCLC); A Systematic Review and Meta-Analysis

**DOI:** 10.1371/journal.pone.0107677

**Published:** 2014-09-18

**Authors:** Anastasios Dimou, Lemuel Non, Young Kwang Chae, William J. Tester, Konstantinos N. Syrigos

**Affiliations:** 1 Department of Medicine, Albert Einstein Medical Center, Philadelphia, Pennsylvania, United States of America; 2 Northwestern Medicine Developmental Therapeutics Institute (NMDTI), Northwestern University Feinberg School of Medicine, Robert H. Lurie Comprehensive Cancer Center of Northwestern University, Chicago, Illinois, United States of America; 3 Albert Einstein Healthcare Network, Cancer Center, Albert Einstein Medical Center, Philadelphia, Pennsylvania, United States of America; 4 Sotiria General Hospital Cancer Center, The University of Athens, Athens, Greece; H. Lee Moffitt Cancer Center & Research Institute, United States of America

## Abstract

**Objectives:**

MET is a receptor present in the membrane of NSCLC cells and is known to promote cell proliferation, survival and migration. *MET* gene copy number is a common genetic alteration and inhibition o MET emerges as a promising targeted therapy in NSCLC. Here we aim to combine in a meta-analysis, data on the effect of high *MET* gene copy number on the overall survival of patients with resected NSCLC.

**Methods:**

Two independent investigators applied parallel search strategies with the terms “MET AND lung cancer”, “MET AND NSCLC”, “*MET* gene copy number AND prognosis” in PubMed through January 2014. We selected the studies that investigated the association of *MET* gene copy number with survival, in patients who received surgery.

**Results:**

Among 1096 titles that were identified in the initial search, we retrieved 9 studies on retrospective cohorts with adequate retrievable data regarding the prognostic impact of *MET* gene copy number on the survival of patients with NSCLC. Out of those, 6 used FISH and the remaining 3 used RT PCR to assess the *MET* gene copy number in the primary tumor. We calculated the I^2^ statistic to assess heterogeneity (I^2^ = 72%). *MET* gene copy number predicted worse overall survival when all studies were combined in a random effects model (HR = 1.78, 95% CI 1.22–2.60). When only the studies that had at least 50% of adenocarcinoma patients in their populations were included, the effect was significant (five studies, HR 1.55, 95% CI 1.23–1.94). This was not true when we included only the studies with no more than 50% of the patients having adenocarcinoma histology (four studies HR 2.18, 95% CI 0.97–4.90).

**Conclusions:**

Higher *MET* gene copy number in the primary tumor at the time of diagnosis predicts worse outcome in patients with NSCLC. This prognostic impact may be adenocarcinoma histology specific.

## Introduction

Non-Small Cell Lung Cancer (NSCLC) is the leading cause of cancer related death worldwide [1]. Current treatment for metastatic disease is largely dependent on companion analysis of tissue and consists of targeted biologic therapy when a driver mutation is present or conventional chemotherapy at the absence of a putative target [2]–[4]. Sensitizing mutations in the tyrosine kinase domain of the epidermal growth factor receptor (EGFR) or translocations involving the anaplastic lymphoma kinase (ALK) and ROS1 predict response to EGFR tyrosine kinase inhibitors (tkis) and ALK inhibitors respectively. On the other hand, as clinical benefit from chemotherapy with platinum doublets has reached a plateau, it becomes evident that further identification of putative targets and the optimization of targeted therapy strategies holds the premise of improvement in clinical outcomes.

The MET receptor has been characterized as a transmembrane receptor that is activated by the Hepatocyte Growth Factor (HGF). In NSCLC MET can be present in treatment naïve cases [5] but it can also emerge after treatment with TKIs where it mediates secondary resistance to EGFR inhibition [6]. The MET receptor leads to proliferation and inhibition of apoptosis with activation of downstream PI3K/AKT and ERK pathways [7], [8] while it promotes metastasis via the STAT system of transcription factors [9], [10]. Given the significance of MET in the biology of NSCLC, certain MET specific inhibitors have been suggested as potential therapies with promising initial results. Tivantinib, a tyrosine kinase inhibitor in combination with erlotinib was found to prolong progression free survival (PFS) when compared to erlotinib alone in a phase II study in patients with non-squamous NSCLC [11]. On the other hand the combination of erlotinib with onartuzumab, a MET-specific monoclonal antibody prolonged PFS and OS compared with erlotinib alone in patients with MET-positive NSCLC [12].

Gene copy amplification is a common mechanism of MET overexpression and it can be detected with reverse transcriptase polymerase chain reaction (RT PCR) or fluorescence in situ hybridization (FISH). A number of studies have explored the prognostic role of *MET* gene copy amplification in NSCLC. In this systematic review and meta-analysis, we aim to quantitatively review the effect of high vs. low *MET* gene copy number on the overall survival of patients with resected NSCLC.

## Methods

### Selection criteria

We included studies which assessed *MET* gene copy number with RT PCR, FISH or FISH equivalent in patients with NSCLC who underwent surgical resections. In addition, only the studies which reported the hazards ratio (HR) of increased *MET* gene copy number on survival or provided adequate data to calculate the HR were included. We excluded studies not published in full in the English language.

### Search strategy and study identification

We entered the terms “met AND lung cancer”, “met AND NSCLC”, “*MET* gene copy number AND prognosis” in MEDLINE through January 2014. Two authors (A.D. and L.N.) reviewed the abstracts from the search and selected the eligible studies for full publication review. In the second phase of the review, the same authors selected the studies which meet all the study eligibility criteria. In addition, we included in the search all the bibliographies of the identified studies. In case of disagreement, the study was included or excluded on the basis of consensus by all the contributors. In the case of publications with overlapping populations, only the publication with more complete data was included.

### Data extraction

Two authors (A.D. and L.N.) extracted the HR for overall survival with the accompanying 95% CI as the outcome measure from the identified studies. In addition relevant clinicopathologic data including the number of participants, the years of diagnosis, the year of publication, the histologic types, the TNM stage, the median age, the race and gender of the participants were collected. We noted patients who were initially included in individual studies but not in the final analysis. We reviewed the methods section in every included study and noted the exact assay of MET gene copy number assessment as well as the threshold of high MET gene copy number detection.

### Assessment of data quality

Every included study was given a quality score on the basis of a quality assessment tool originally described in the study by Steels et al [13]. Briefly, all contributors evaluated each individual study in every of four domains including scientific design, description of methods, analysis of the data and generalizability of the results. Every category received a score of 0–10 and scores were added to a maximum score of 40. The final score was calculated as a percentage ranging 0–100% with higher scores suggesting better data quality.

### Statistical analysis

A forest plot was utilized to aggregate HRs from the univariate analysis of single studies in a summary HR of the effect of high *MET* gene copy number on survival. The I^2^ test was used to assess heterogeneity between studies. In the case of I^2^>50%, the summary HR and the accompanying 95% CI were calculated with a random effects model whereas we used a fixed effects model in the case of low heterogeneity as defined by I^2^≤50%. We looked for publication bias with the aid of a funnel plot. Statistical significance was defined at the level of 0.05. All statistics were performed with RevMan 5.2.8 downloaded from the Cochrane website (http://tech.cochrane.org/revman/download).

## Results

### Study selection plot

**Figure 1:**
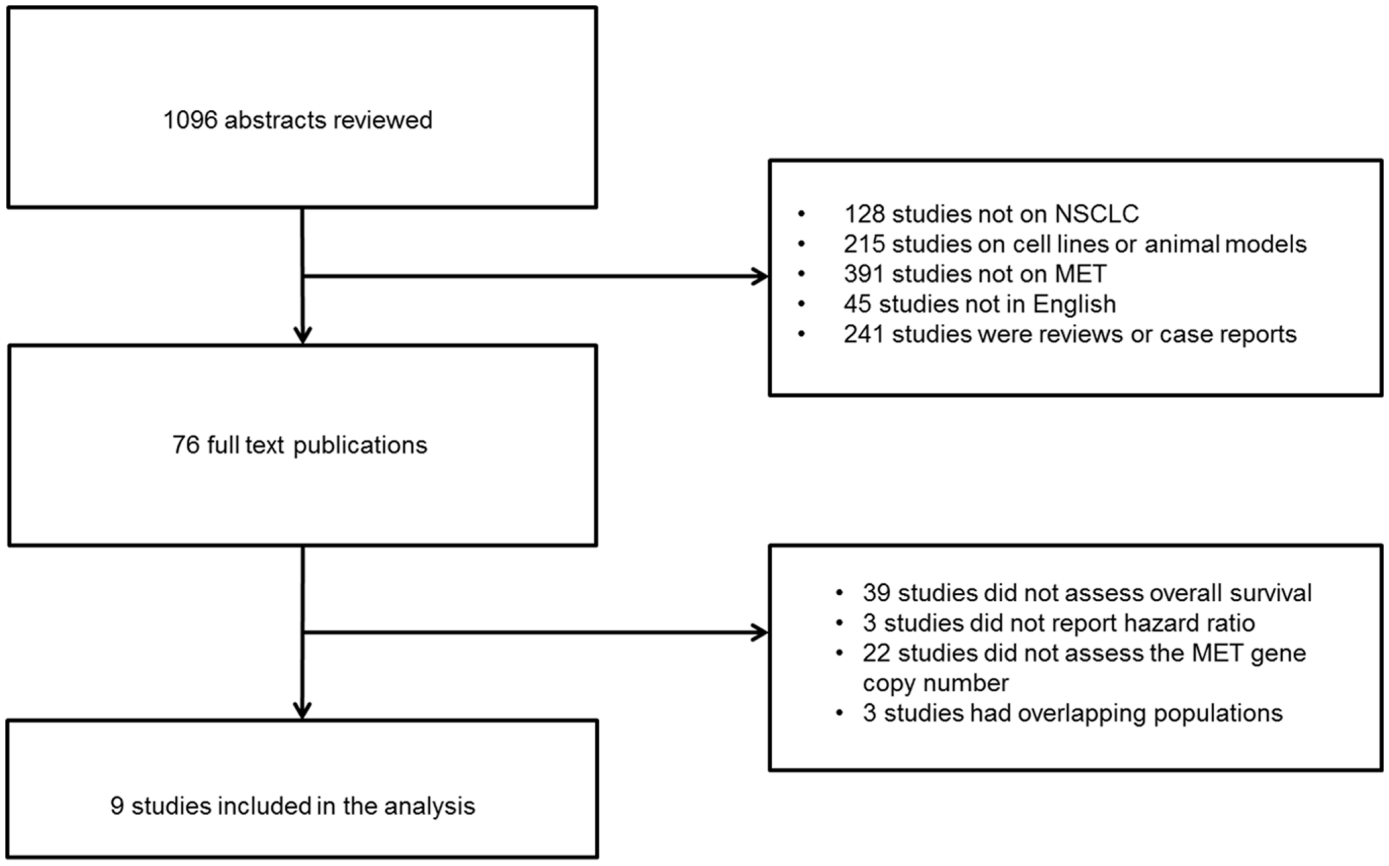
Flow chart illustrating the search process and the reasons for excluding studies.

Our initial search identified 1096 titles. After reviewing the corresponding abstracts we excluded 1020 publications for being reviews or case reports, for focusing on cell lines or non-human subjects, not being relevant to the MET receptor, not being relevant to NSCLC, or on the basis of language criteria (figure 1). After the initial review we identified 76 studies for which we performed full text review. From this subset of studies we selected 9 to be included in the summary analysis after excluding 67 for not including overall survival analysis, not assessing the gene copy number, not reporting the hazards ratio and containing overlapping data (figure 1). We found three studies which performed survival analysis of high *MET* gene copy number tumors but did not report the HR [14]–[16]. Jin et al [15] reported data on 141 surgically resected stage I adenocarcinomas; they found that higher MET gene copy number is associated with worse overall survival (p = 0.01) and progression free survival (p<0.001), nevertheless there was no HR reported. On the other hand, Tanaka et al [16] studied *MET* gene copy number in 136 surgically resected lung adenocarcinomas and used both PathVysion and the Cappuzo system to define high and low *MET* gene copy number. With the former methodology, the investigators identified a worse overall survival with higher *MET* gene copy number (p = 0.03), while with the latter methodology no association was found between *MET* gene copy number and overall survival (p = 0.22). Finally, Okuda et al [14] studied *MET* gene copy number with RT PCR in an Asian population of 213 patients and by using a cut point of three *MET* gene copies reported a shorter overall survival in patients with higher gene copy number.

### Study characteristics

We identified a total of nine studies [17]–[25] fulfilling all the eligibility criteria to be included in the meta analysis (table 1). There were six studies in Asian and three in non-Asian populations, four studies included cases diagnosed before 2004 only, three studies assessed the *MET* gene copy number with RT PCR, four with FISH and two with silver in situ hybridization (SISH). SISH utilizes light microscopy instead of fluorescence enabling the concomitant evaluation of gene copy number and cell morphology while at the same time allows for signal preservation for a longer time compared with FISH. Quality analysis revealed that all studies but one had comparable quality scores. In summary, most of the studies adequately described the eligibility criteria for the study population, included patient demographics, revealed the source of the tissue, described the method of *MET* gene copy number assessment, the study design, the objectives and the *MET* gene copy number positivity cut off (Checklist S1). Limitations to study quality included observational design, lack of blinding, lack of positive and negative controls, lack of information on missing cases and tissue handling (Table S1). Go et al [24] reported the hazards ratio in patients with squamous cell histology only (n = 97 out of the initial cohort of 180 patients).

**Table 1:** Study characteristics.

Study	N	Ethnicity	Year of diagnosis	Quality score	% AC	% stage I	Method
Capuzzo et al [21]	376	Non Asian	2000–2004	77.5%	53	37	FISH
Chen et al [22]	97	Asian	1996–1998	72.5%	0	MD	RT PCR
Dziadziuszco et al [23]	140	Non Asian	MD	75%	29	39	FISH
Go et al [24]	180	Asian	1995–2000	72.5%	40	24	FISH
Kanteti et al [17]	23	Non Asian	MD	47.5%	100	MD	RT PCR
Park et al [18]	380	Asian	1994–2001	80%	35	50	FISH
Sun et al [19]	61	Asian	2004–2008	77.5%	46	MD	RT PCR
Tachibana et al [25]	106	Asian	2001–2008	75%	100	90	FISH
Tsuta et al [20]	906	Asian	1997–2007	75%	75	47	FISH

MD = missing data, AC = adenocarcinoma.

### Survival analysis

**Figure 2:**
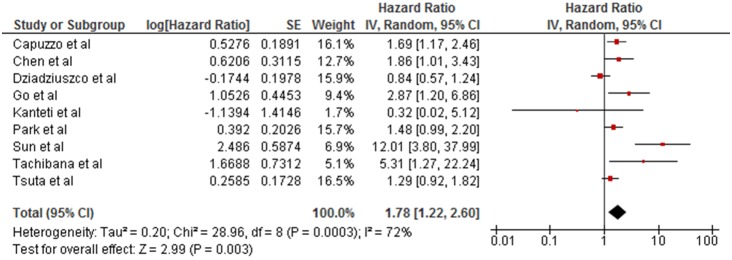
Forest plot combining all the included studies hazard ratio in a cumulative hazard ratio with a random effects model.

We then quantified the summary effect of high *MET* gene copy number on patient survival with the use of a random effect model as shown in the forest plot of figure 2. Heterogeneity across all studies is statistically significant (I^2^ = 72%, p = 0.0003) and the net HR and 95% CI is 1.78 (1.22–2.60) indicating a worse overall survival with higher *MET* gene copy number.

### Sensitivity analysis

We tried to explain the observed heterogeneity with regards to different population characteristics in different studies. We found a negative net effect of *MET* gene copy number on survival in studies with Asian populations (HR = 2.31 95% CI 1.31–3.79), in studies where the reported histology was adenocarcinoma in more than half of the cases (HR = 1.55 95% CI 1.23–1.94), in studies which used FISH (HR = 1.48 95% CI 1.06–2.06) and in studies where the patients were diagnosed before 2004 (HR = 1.70 95% CI 1.34–2.16) (table 2). In contrast there was no statistical significant net effect of the *MET* gene copy number on overall survival in studies with non-Asian populations, studies where adenocarcinoma histology was reported in less than half of the cases and in studies where RT PCR was used to assess the *MET* gene copy number (table 2).

**Table 2:** 

Additional criteria	N	HR (95% CI)	I^2^ (p value for I^2^)
Quality score ≥70%	8	1.83 (1.25–2.68)	75% (0.0002)
Asian	6	2.31 (1.31–3.79)	72% (0.003)
Non Asian	3	1.12 (0.58–2.16)	73% (0.02)
AC%≥50%	5	1.55 (1.23–1.94)	30% (0.22)
AC%<50%	4	2.18 (0.97–4.90)	87% (<0.0001)
FISH	6	1.48 (1.06–2.06)	64% (0.02)
RT PCR	3	2.63 (0.51–13.54)	80% (0.006)
Year of diagnosis <2004	4	1.70 (1.34–2.16)	0% (0.59)

### Publication bias

**Figure 3:**
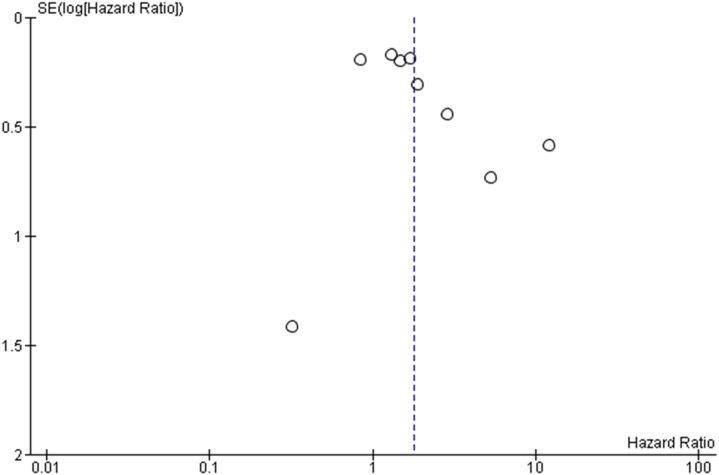
Funnel plot.

Finally, we attempted to estimate the risk for publication bias with a funnel plot (figure 3). Although there appears to be a correlation between the study size and the effects estimate which adds some asymmetry to the plot, there are four studies that do not reach statistical significance.

## Discussion

In the current study we reviewed the literature on *MET* gene copy number and the potential association with survival in patients with surgically resected NSCLC. We further combined the available data in a meta-analysis and found a cumulative hazards ratio of 1.78 indicating worse overall survival with higher *MET* gene copy number in this patient population. In the subgroup analysis Asian ethnicity, FISH methodology, adenocarcinoma histology and year of diagnosis earlier than 2004 were indicators of a negative effect of MET gene copy number on survival. We assessed heterogeneity across different studies with the I^2^ statistic and found significant heterogeneity when all studies were included in the analysis. Interestingly, heterogeneity was low among studies including mostly adenocarcinoma cases and among studies including patients diagnosed before 2004.

The negative effect of higher *MET* gene copy number on the overall survival of patients with NSCLC can be explained by the role of MET in cancer biology. Among the different downstream pathways activated by MET, the JAK/STAT cascade is of particular importance for promoting cell motility, migration and metastasis [26], [27]. In our study, only studies on patients who received surgery were involved. Unfortunately a considerable number of these patients will relapse despite an R0 resection and will finally die of their disease. It is possible that patients, who relapse after an R0 resection, have higher metastatic potential and therefore occult metastases at the time of surgery. In this context, higher *MET* gene copy number might identify a subgroup of patients with higher metastatic potential.

There are a number of plausible explanations for the results of the sensitivity analysis in the present meta-analysis. First, studies that include patients diagnosed before 2004 were published earlier introducing a potential time lag bias in the interpretation of the data [28]. Alternatively, adjuvant chemotherapy was established after 2004 for patients after resection in the case of stage II, III, or IB with adverse prognostic factors [29]. The potential use of adjuvant chemotherapy in patients diagnosed after 2004 might have confounded the prognostic significance of *MET* gene copy number in this subset of studies and increased heterogeneity. Additionally, dual positivity for *MET* gene amplification and *EGFR* mutations might confound results after 2004 in case of treatment with an EGFR TKI inhibitor after disease progression. The effect of dual positivity for *MET* gene amplification and *EGFR* mutations might account for the different effects of increased *MET* gene copy number on prognosis between Asian and non-Asian populations. It is possible that different effect of high *MET* gene copy number on survival in patient populations with distinct clinicopathologic characteristics might reflect differences in other pathways which modify the MET pathway.

It is not clear how the biology of MET receptor differs between adenocarcinoma and squamous cell carcinoma in NSCLC. A possibility is that additional histotype specific genetic alterations might interfere with the net effect of MET, or regulate MET expression. Krishnaswamy et al [30] reported the presence of germline mutations in the semaphorin portion of the MET receptor occurring preferably in Asian populations and squamous cell carcinomas. The presence of N375S, the most common mutation in this group of mutations reduces the affinity of the MET receptor to hepatocyte growth factor and is associated with resistance to MET inhibition with the MET inhibitor SU11274. It is largely unknown whether such mutations or other MET regulation molecules account for the observed difference in the association between high *MET* gene copy number and survival.

Inhibition of MET is currently feasible small tyrosine kinase inhibitors like tivantinib or with monoclonal antibodies like onartuzumab. Tivantinib as shown in the MARQUEE trial [31], [32] prolongs progression free survival but not overall survival when combined with erlotinib compared to erlotinib alone in the pretreated unselected population with non-squamous NSCLC. A subgroup analysis in MET-positive by immunohistochemistry patients showed benefit in both the progression free survival and overall survival. In a similar fashion, the MetLung study [33] compared onartuzumab in combination with erlotinib compared to erlotinib alone in the MET-positive by immunohistochemistry pretreated population of patients with NSCLC regardless of histologic type. Taking into account the results of the present meta-analysis it will be interesting to see whether the potential benefit from onartuzumab is present in all the MET positive patients or restricted to MET positive patients with adenocarcinoma only. The MetLung study was closed prematurely based on the data from the interim analysis which showed no difference in overall survival which was the primary outcome [33]. Interestingly, it was recently presented in 2014 ASCO Annual meeting that crizotinib demonstrated efficacy in patients with FISH proven *MET* amplification in a phase I trial [34]. These two studies generate the hypothesis that *MET* amplification status might be a better predictive biomarker for MET targeted therapies compared to protein expression by immunohistochemistry. We identified significant heterogeneity when all the identified studies are included in the meta-analysis. Possible reasons for heterogeneity might be populations with distinct demographics, possible interference of *MET* gene copy number prognostic impact and adjuvant chemotherapy as well as pre-analytical and analytical variance across different studies. For example, the cut point for high *MET* gene copy number ranges between 3 and 5, whereas some studies use in situ hybridization and others RT PCR. Additionally, the use of archived tissue poses a potential risk for loss of signal in FISH and RT PCR assays. Interestingly, we found that the studies where most of the patients were of adenocarcinoma histology were relatively homogeneous as were studies in which patients were diagnosed before 2004.

There are a number of limitations in this meta-analysis. First, it is a meta-analysis of retrospective studies and therefore carries the biases of the retrospective design of the individual studies. Second, the number of studies is small, increasing the type I error. In addition, we observed an asymmetry pattern at the funnel plot. Asymmetry in funnel plots is illustrated by a correlation between the sample size and an estimate of the outcome effect and it indicates the presence of publication bias, heterogeneity or alternatively can be the result of random error [28]. In our meta-analysis there is a considerable proportion of studies which report no statistical significant results (four out of nine). If only publication bias accounts for the funnel plot asymmetry, one would expect more studies showing an association of *MET* gene copy number with overall survival, either a positive or negative. Therefore, we believe that heterogeneity across studies accounts partially for the observed asymmetry. Finally, there were three studies which were not involved in the meta-analysis because no hazards ratio was reported [14]–[16]. Nevertheless, it is unlikely that omission of these studies has altered the cumulative effect of high *MET* gene copy number on survival as they both report a positive significant effect on overall survival and they both include mostly or only patients with adenocarcinoma histology.

In conclusion, here we present a meta-analysis of nine retrospective studies which analyze the prognostic effect of *MET* gene copy number in NSCLC. We suggest that the worse overall survival observed in patients with higher gene copy number might be histotype specific and generates the hypothesis that patients with adenocarcinoma and high *MET* gene copy numbermight potentially benefit from adjuvant chemotherapy after resection.

## Supporting Information

Table S1 (XLSX)

Checklist S1 **PRISMA Checklist.**
(DOC)

## References

[1] Jemal A, Bray F (2011) Center MM, Ferlay J, Ward E, et al (2011) Global cancer statistics. CA Cancer J Clin 61: 69–90 10.3322/caac.20107 21296855

[2] Hirsch FR, Janne PA, Eberhardt WE, Cappuzzo F, Thatcher N, et al (2013) Epidermal growth factor receptor inhibition in lung cancer: status 2012. J Thorac Oncol 8: 373–384 10.1097/JTO.0b013e31827ed0ff 23370315

[3] Sasaki T, Janne PA (2011) New strategies for treatment of ALK-rearranged non-small cell lung cancers. Clin Cancer Res 17: 7213–7218 10.1158/1078-0432.CCR-11-1404 22010214 PMC3477548

[4] Langer CJ, Besse B, Gualberto A, Brambilla E, Soria JC (2010) The evolving role of histology in the management of advanced non-small-cell lung cancer. J Clin Oncol 28: 5311–5320 10.1200/JCO.2010.28.8126 21079145

[5] Turke AB, Zejnullahu K, Wu YL, Song Y, Dias-Santagata D, et al (2010) Preexistence and clonal selection of MET amplification in EGFR mutant NSCLC. Cancer Cell 17: 77–88 10.1016/j.ccr.2009.11.022 20129249 PMC2980857

[6] Engelman JA, Zejnullahu K, Mitsudomi T, Song Y, Hyland C, et al (2007) MET amplification leads to gefitinib resistance in lung cancer by activating ERBB3 signaling. Science 316: 1039–1043 10.1126/science.1141478 17463250

[7] Graziani A, Gramaglia D, dalla Zonca P, Comoglio PM (1993) Hepatocyte growth factor/scatter factor stimulates the Ras-guanine nucleotide exchanger. J Biol Chem 268: 9165–9168. PMID: 8387483. 8387483

[8] Matsumoto K, Nakamura T (2006) Hepatocyte growth factor and the Met system as a mediator of tumor-stromal interactions. Int J Cancer 119: 477–483 10.1002/ijc.21808 16453287

[9] Zhang YW, Wang LM, Jove R, Vande Woude GF (2002) Requirement of Stat3 signaling for HGF/SF-Met mediated tumorigenesis. Oncogene 21: 217–226 10.1038/sj/onc/1205004 11803465

[10] Boccaccio C, Ando M, Tamagnone L, Bardelli A, Michieli P, et al (1998) Induction of epithelial tubules by growth factor HGF depends on the STAT pathway. Nature 391: 285–288 10.1038/34657 9440692

[11] Sequist LV, von Pawel J, Garmey EG, Akerley WL, Brugger W, et al (2011) Randomized phase II study of erlotinib plus tivantinib versus erlotinib plus placebo in previously treated non-small-cell lung cancer. J Clin Oncol 29: 3307–3315 10.1200/JCO.2010.34.0570 21768463

[12] Spigel DR, Ervin TJ, Ramlau RA, Daniel DB, Goldschmidt JH Jr, et al (2013) Randomized phase II trial of Onartuzumab in combination with erlotinib in patients with advanced non-small-cell lung cancer. J Clin Oncol 31: 4105–4114 10.1200/JCO.2012.47.4189 24101053 PMC4878106

[13] Steels E, Paesmans M, Berghmans T, Branle F, Lemaitre F, et al.. (2001) Role of p53 as a prognostic factor for survival in lung cancer: a systematic review of the literature with a meta-analysis. Eur Respir J 18: 705–719. PMID: 11716177. 10.1183/09031936.01.00062201 11716177

[14] Okuda K, Sasaki H, Yukiue H, Yano M, Fujii Y (2008) Met gene copy number predicts the prognosis for completely resected non-small cell lung cancer. Cancer Sci 99: 2280–2285 10.1111/j.1349-7006.2008.00916.x 19037978 PMC11159911

[15] Jin Y, Sun PL, Kim H, Seo AN, Jheon S, et al.. (2013) MET Gene Copy Number Gain is an Independent Poor Prognostic Marker in Korean Stage I Lung Adenocarcinomas. Ann Surg Oncol. doi:10.1245/s10434-013-3355-1. 10.1245/s10434-013-3355-1 24212721

[16] Tanaka A, Sueoka-Aragane N, Nakamura T, Takeda Y, Mitsuoka M, et al (2012) Co-existence of positive MET FISH status with EGFR mutations signifies poor prognosis in lung adenocarcinoma patients. Lung Cancer 75: 89–94 10.1016/j.lungcan.2011.06.004 21733594

[17] Kanteti R, Yala S, Ferguson MK, Salgia R (2009) MET, HGF, EGFR, and PXN gene copy number in lung cancer using DNA extracts from FFPE archival samples and prognostic significance. J Environ Pathol Toxicol Oncol 28: 89–98. PMID: 19817696. 10.1615/jenvironpatholtoxicoloncol.v28.i2.10 PMC2761881 19817696

[18] Park S, Choi YL, Sung CO, An J, Seo J, et al.. (2012) High MET copy number and MET overexpression: poor outcome in non-small cell lung cancer patients. Histol Histopathol 27: 197–207. PMID: 22207554. 10.14670/HH-27.197 22207554

[19] Sun W, Song L, Ai T, Zhang Y, Gao Y, et al (2013) Prognostic value of MET, cyclin D1 and MET gene copy number in non-small cell lung cancer. J Biomed Res 27: 220–230 10.7555/JBR.27.20130004 23720678 PMC3664729

[20] Tsuta K, Kozu Y, Mimae T, Yoshida A, Kohno T, et al (2012) c-MET/phospho-MET protein expression and MET gene copy number in non-small cell lung carcinomas. J Thorac Oncol 7: 331–339 10.1097/JTO.0b013e318241655f 22198430

[21] Cappuzzo F, Marchetti A, Skokan M, Rossi E, Gajapathy S, et al (2009) Increased MET gene copy number negatively affects survival of surgically resected non-small-cell lung cancer patients. J Clin Oncol 27: 1667–1674 10.1200/JCO.2008.19.1635 19255323 PMC3341799

[22] Chen YT, Chang JW, Liu HP, Yu TF, Chiu YT, et al (2011) Clinical implications of high MET gene dosage in non-small cell lung cancer patients without previous tyrosine kinase inhibitor treatment. J Thorac Oncol 6: 2027–2035 10.1097/JTO.0b013e3182307e92 22052229

[23] Dziadziuszko R, Wynes MW, Singh S, Asuncion BR, Ranger-Moore J, et al (2012) Correlation between MET gene copy number by silver in situ hybridization and protein expression by immunohistochemistry in non-small cell lung cancer. J Thorac Oncol 7: 340–347 10.1097/JTO.0b013e318240ca0d 22237262 PMC3358920

[24] Go H, Jeon YK, Park HJ, Sung SW, Seo JW, et al (2010) High MET gene copy number leads to shorter survival in patients with non-small cell lung cancer. J Thorac Oncol 5: 305–313 10.1097/JTO.0b013e3181ce3d1d 20107422

[25] Tachibana K, Minami Y, Shiba-Ishii A, Kano J, Nakazato Y, et al (2012) Abnormality of the hepatocyte growth factor/MET pathway in pulmonary adenocarcinogenesis. Lung Cancer 75: 181–188. 21872356 10.1016/j.lungcan.2011.07.008

[26] Song L, Turkson J, Karras JG, Jove R, Haura EB (2003) Activation of Stat3 by receptor tyrosine kinases and cytokines regulates survival in human non-small cell carcinoma cells. Oncogene 22: 4150–4165 10.1016/j.lungcan.2011.07.008 12833138

[27] Franci C, Zhou J, Jiang Z, Modrusan Z, Good Z, et al (2013) Biomarkers of residual disease, disseminated tumor cells, and metastases in the MMTV-PyMT breast cancer model. PLoS One 8: e58183 10.1371/journal.pone.0058183 23520493 PMC3592916

[28] Sterne JA, Sutton AJ, Ioannidis JP, Terrin N, Jones DR, et al (2011) Recommendations for examining and interpreting funnel plot asymmetry in meta-analyses of randomised controlled trials. BMJ 343: d4002 10.1136/bmj.d4002 21784880

[29] Hotta K, Matsuo K, Ueoka H, Kiura K, Tabata M, et al.. (2004) Role of adjuvant chemotherapy in patients with resected non-small-cell lung cancer: reappraisal with a meta-analysis of randomized controlled trials. J Clin Oncol 22: 3860–3867. PMID: 15326194. 10.1200/JCO.2004.01.153 15326194

[30] Krishnaswamy S, Kanteti R, Duke-Cohan JS, Loganathan S, Liu W, et al (2009) Ethnic differences and functional analysis of MET mutations in lung cancer. Clin Cancer Res 15: 5714–5723 10.1158/1078-0432.CCR-09-0070 19723643 PMC2767337

[31] Scagliotti GV, Novello S, Schiller JH, Hirsh V, Sequist LV, et al (2012) Rationale and design of MARQUEE: a phase III, randomized, double-blind study of tivantinib plus erlotinib versus placebo plus erlotinib in previously treated patients with locally advanced or metastatic, nonsquamous, non-small-cell lung cancer. Clin Lung Cancer 13: 391–395 10.1016/j.cllc.2012.01.003 22440336

[32] Scagliotti G, Novello S, Ramlau R, Favaretto A, Barlesi F, et al.. (2013) MARQUEE: A Randomized, Double-Blind, Placebo-Controlled, Phase 3 Trial of Tivantinib (ARQ 197) Plus Erlotinib Versus Placebo Plus Erlotinib in Previously Treated Patients With Locally Advanced or Metastatic, Non-Squamous, Non-Small-Cell Lung Cancer (NSCLC); 2013; Amsterdam, Netherlands.

[33] Spigel DR, Edelman MJ, Mok T, O’Byrne K, Paz-Ares L, et al (2012) Treatment Rationale Study Design for the MetLung Trial: A Randomized, Double-Blind Phase III Study of Onartuzumab (MetMAb) in Combination With Erlotinib Versus Erlotinib Alone in Patients Who Have Received Standard Chemotherapy for Stage IIIB or IV Met-Positive Non-Small-Cell Lung Cancer. Clin Lung Cancer 13: 500–504 10.1016/j.cllc.2012.05.009 23063071

[34] Camidge D, Ou S, Shapiro G, Otterson G, Villaruz L, et al.. (2014) Efficacy and safety of crizotinib in patients with advanced c-MET-amplified non-small cell lung cancer (NSCLC). J Clin Oncol 32: 5s, 2014 (suppl; abstr 8001).

